# Diagnostic dilemmas: a multi-institutional retrospective analysis of adrenal incidentaloma pathology based on radiographic size

**DOI:** 10.1186/s12894-022-01024-5

**Published:** 2022-04-30

**Authors:** David Zekan, Robert Scott King, Ali Hajiran, Apexa Patel, Samuel Deem, Adam Luchey

**Affiliations:** 1 Department of Urology, West Virginia University, 1 Medical Center Drive, Morgantown, WV 26505 USA; 2 Department of Urology, Charleston Area Medical Center, 3100 MacCorkle Ave SE Suite 602, Charleston, WV 25304 USA; 3 Health Education and Research Institute, Charleston Area Medical Center, 3110 MacCorkle Ave SE, Charleston, WV 25304 USA

**Keywords:** Cross-sectional imaging, Adrenocortical carcinoma, Malignancy, CT, MRI

## Abstract

**Introduction/background:**

Adrenal incidentalomas (AIs) are masses > 1 cm found incidentally during radiographic imaging. They are present in up to 4.4% of patients undergoing CT scan, and incidence is increasing with usage and sensitivity of cross-sectional imaging. Most result in diagnosis of adrenal cortical adenoma, questioning guidelines recommending removal of all AIs with negative functional workup. This retrospective study analyzes histological outcome based on size of non-functional adrenal masses.

**Material and methods:**

10 years of data was analyzed from two academic institutions. Exclusion criteria included patients with positive functional workups, those who underwent adrenalectomy during nephrectomy, < 18 years, and incomplete records. AI radiologic and histologic size, histologic outcome, laterality, imaging modality, gender, and age were collected. T-test was used for comparison of continuous variables, and the two-sided Fisher’s exact or chi-square test were used to determine differences for categorical variables. Univariate analysis of each independent variable was performed using simple logistic regression.

**Results:**

73 adrenalectomies met the above inclusion criteria. 60 were detected on CT scan, 12 on MRI, and one on ultrasound. Eight of 73 cases resulted in malignant pathology, 3 of which were adrenocortical carcinoma (ACC). Each ACC measured > 6 cm, with mean radiologic and pathologic sizes of 11.2 cm and 11.3 cm. Both radiologic and pathologic size were significant predictors of malignancy (*p* = 0.008 and 0.011).

**Conclusions:**

Our results question the generally-accepted 4 cm cutoff for excision of metabolically-silent AIs. They suggest a 6 cm threshold would suffice to avoid removal of benign lesions while maintaining sensitivity for ACC.

## Introduction

The malignant potential of a small, incidentally found lesion of the adrenal gland causes great anxiety amongst patients, but rarely causes harm. An adrenal incidentaloma (AI) is defined as mass measuring greater than 1 cm on the adrenal gland found fortuitously during radiographic imaging [1]. The diameter of the average AI measures approximately 3–3.5 cm on Computed Tomography (CT) scan [2]. With increased utilization and advancements in the sensitivity of medical imaging, adrenal masses are observed in as many as 4.4% of all patients undergoing CT scan [3].

Over the past two decades, an increasing body of evidence has supported restructuring the current guidelines for the management of AIs. Most AIs result in the histological diagnosis of benign adrenal cortical adenoma [2]. Adrenocortical carcinoma (ACC) is more commonly diagnosed at younger ages than benign adrenal masses and is found more often in males than females [2]. For patients without a history of malignancy, the estimated prevalence of ACC is less than 2 cases per million adrenal lesions identified [4]. Twenty-five percent of benign masses increase in size during follow up; however, given the estimated 1 in 1000 risk of malignant transformation over time for benign masses, continued observation may be an appropriate option [2]. Exposing patients to additional risks with surgery for a rare chance of cancer in smaller adrenal masses may not be warranted.

The American Association of Clinical Endocrinologists (AACE) and American Association of Endocrine Surgeons (AAES) guidelines have changed over the last decade in regards to excision based on size from a previous cutoff of ≥ 6 cm to now only > 4 cm [5]. Current clinical guidelines recommend a workup to include contrasted cross-sectional imaging and a functional evaluation to investigate for hormonal activity. Surgical excision is then recommended if the lesion is radiographically suspicious, > 4 cm in size, or hormonally active [6]. Due to the rarity of ACC, most of these recommendations are based on information obtained from controlled trials without randomization (Level 3 Evidence) [5].

Because there have been few randomized controlled studies regarding the management of AIs, there is a lack of qualified management guidelines for patient care [6]. To address this clinical question, we sought to perform a collaborative effort reviewing ten years of adrenalectomy data comparing pathologic outcomes based on size criteria. It was hypothesized that our data would further support a needed modification in the current guidelines for removal of AIs.

## Materials and methods

Following approval from respective institutional review boards, including waivers of informed consent from West Virginia University Institutional Review Board and the CAMC/WVU-Charleston Division Institutional Review Board for the Protection of Human Subjects, a two-institution retrospective chart review of adrenalectomies over 10 years was performed. Patients who underwent adrenalectomy at two tertiary care centers, Charleston Area Medical Center (CAMC) and West Virginia University (WVU), from January 1, 2005 through December 31, 2015 were included. Patients who underwent adrenalectomy during nephrectomy, were less than 18 years of age, or had incomplete medical records were excluded. Data analysis was then performed on those with available cross-sectional imaging who underwent adrenalectomy with no history of malignancy and a negative functional workup.

The Center for Health Service and Outcomes Research at CAMC conducted data analysis. For this exploratory study, selection of appropriate statistical measures was determined by CAMC’s Center for Health Education and Research Institute. Two-tailed chi-square alpha was set at 0.05. T-test was used for comparison of continuous variables, and the two-sided Fisher’s exact or chi square test were used to determine differences for categorical variables. Univariate analysis of each independent variable was performed using simple logistic regression. A variable with a univariate *p* < 0.10 was included in the model for multivariate analysis. A *p* value < 0.05 was considered statistically significant.

## Results

A total of 234 patients underwent adrenalectomy from January 1, 2005 to December 31, 2015. 161 cases were excluded: 61 hormonally active lesions (based on pre-operative testing; including subclinical Cushing’s Syndrome), 55 known metastases, 10 adrenalectomies during resection of a primary renal lesion, and 35 with inadequate records or < 18 years of age.

**Fig. 1:**
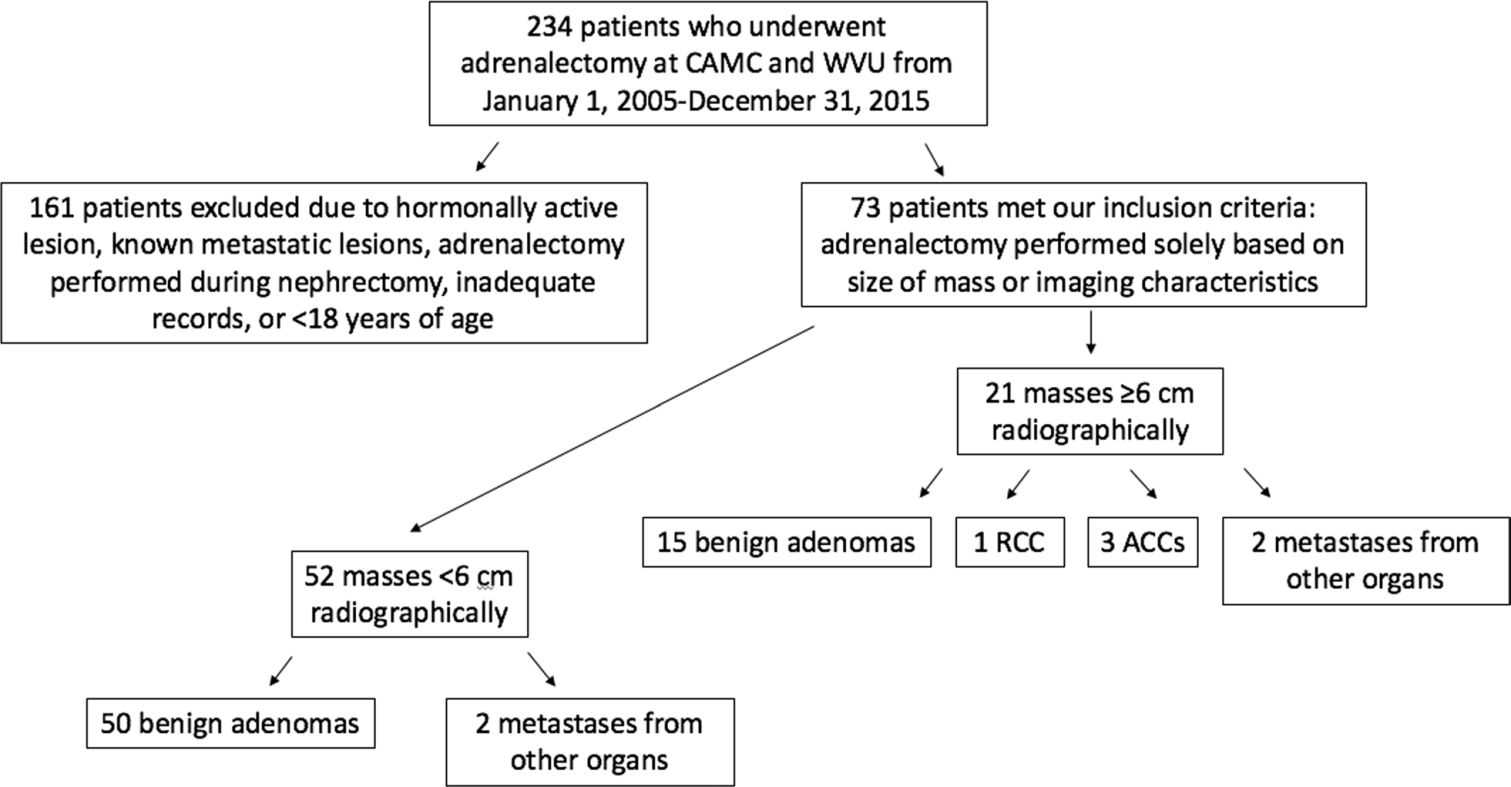
Pathologic breakdown based on radiologic size of adrenal incidentalomas. *RCC* renal cell carcinoma, *ACC* adrenocortical carcinoma

There were 73 adrenalectomies performed based on size criteria and imaging characteristics (contrast uptake and washout) of the adrenal mass (1.7–22.8 cm), as opposed to metabolically active lesions. The average age was 56.8 years with M:F ratio of 0.62:1. One patient in the analyzed group had bilateral masses, while 44 had left-sided masses and 28 had right-sided masses. 60 of the masses were determined appropriate for resection based on CT scan, while 12 were detected on MRI, and one was seen only on ultrasound. In all but 8 of the 73 total adrenalectomies performed based on size criteria alone, final pathology reported benign histology. Three of these cases would result in a histologic diagnosis of ACC, each measuring ≥ 6 cm (the smallest of which measured 7 cm); all (53 total) masses measuring < 6 cm removed solely on the basis of size criteria were benign aside from a single metastasis (Fig. 1).

The ACC mean radiographic and pathologic size was 11.2 cm and 12.3 cm, respectively. ACCs accounted for 14.2% of the 21 adrenalectomies performed for masses ≥ 6 cm.

Thus, ≥ 6 cm was deemed an appropriate cutoff for data analysis for the purposes of this study, as the smallest ACC seen in our cohort was 7 cm and the commonly used cutoff for comparison in the literature is 6 cm [7]. Neither gender nor age were significant predictors of the adrenal mass size category [< 6 cm or ≥ 6 cm; determined both radiologically and pathologically as the two were not always concordant of patients in this study (Table 1)].

**Table 1:** Radiologic and pathologic size discordance amongst adrenal incidentalomas: demonstration of statistically significant discordance between the radiologic (pre-operative) and pathologic (post-operative) sizes of adrenal incidentalomas included in our analysis

Pathologic size (cm)	Radiographic size (cm)		*p* value
	< 6	≥ 6	
< 6	46 (88.46)	1 (4.76)	< 0.0001
≥ 6	6 (11.54)	20 (95.24)	

However, size was predictive of pathology, most importantly malignancy (*p* = 0.008 and 0.011, Table 2).

**Table 2:** Demographics and pathology based on radiologic and pathologic size

	< 6	≥ 6	*p* value
*Radiological size (cm)*			
Male	20 (38.46)	8 (38.10)	0.976
Female	32 (61.54)	13 (61.90)	
Age (years)	59.09 ± 11.81	56.33 ± 11.98	0.390
*Pathologic status based on radiologic size*			
Adrenocortical carcinoma	0	3 (14.29)	0.008*
Kidney cancer	0	1 (4.76)	
Benign adenoma	50 (96.15)	15 (71.43)	
Metastatic from other organ	2 (3.85)	2 (9.52)	
*Pathological size (cm)*			
Male	18 (38.30)	10 (38.46)	0.989
Female	29 (61.70)	16 (61.54)	
Age (years)	58.95 ± 12.01	56.96 ± 11.66	0.494
*Pathologic status based on pathologic size*			
Adrenocortical carcinoma	0	3 (11.54)	0.011*
Kidney cancer	0	1 (3.85)	
Benign adenoma	46 (97.87)	19 (73.08)	
Metastatic from other organ	1 (2.13)	3 (11.54)	

* Statistically significant

Namely, all adrenocortical carcinomas (3), apparent adrenal masses on pre-operative imaging that were found to be renal cell carcinomas pathologically (1), and 50% of adrenal masses that were incidentally found to be metastases (2 of 4) fell into the ≥ 6 cm category. Of the 55 masses resected based on size alone that showed adrenal adenoma as the final pathology, only 19 fell into the ≥ 6 cm category (34.5%) (Fig. 1).

**Fig. 2:**
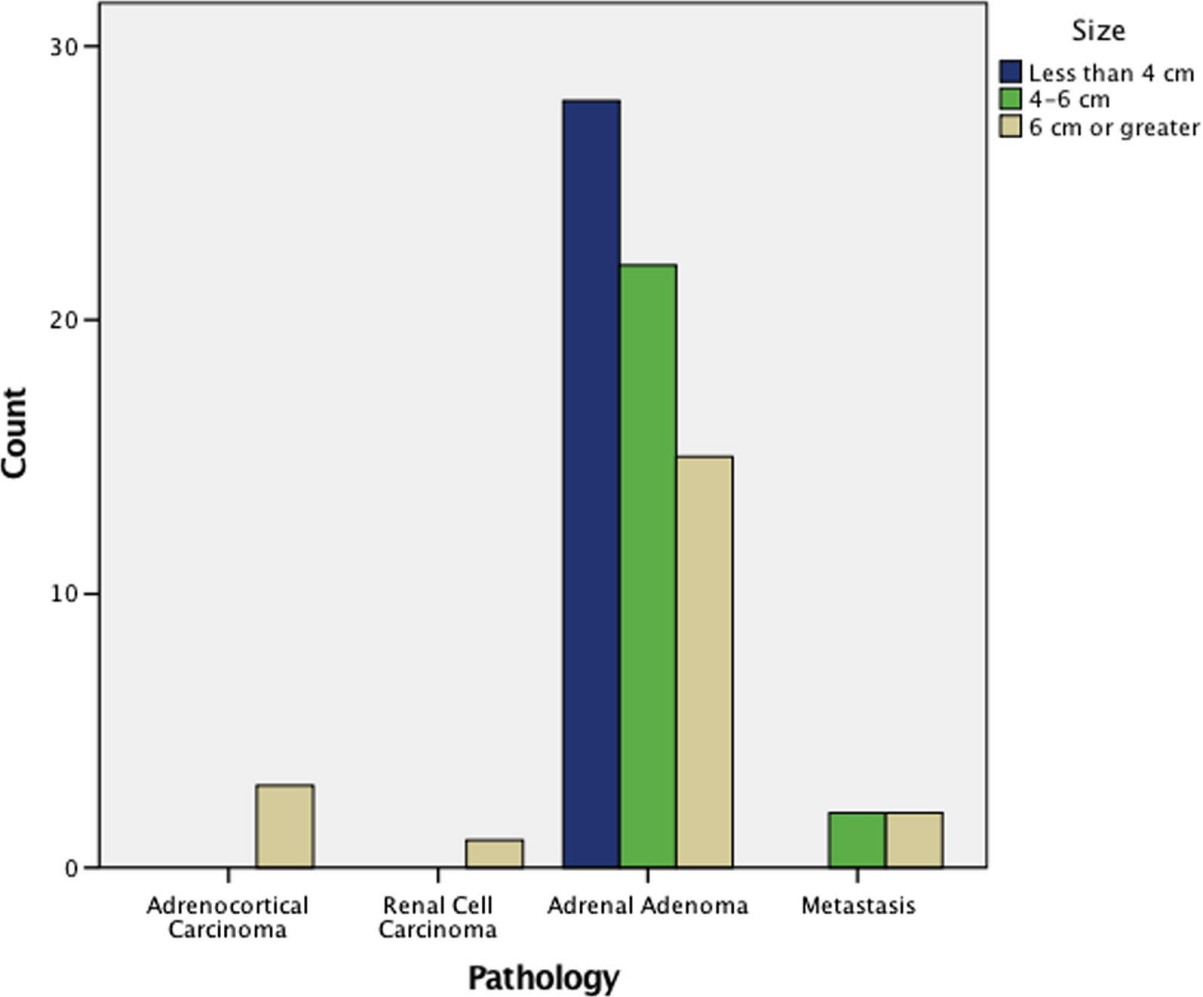
Pathologic breakdown of included adrenal masses based on radiographic size categorized into the guideline-recommended cutoff for resection, this study’s suggested cutoff for resection, and those in-between, respectively

Crosstabulation analysis was also performed to compare the expected and observed number of each pathologic result within the < 4 cm, 4–6 cm, and ≥ 6 cm groups to reinforce our chosen 6 cm cutoff for the above analysis. This further exemplified that a statistically significant higher number than expected of ACCs fell into the ≥ 6 cm category within the above dataset with all ACCs (3) falling into the ≥ 6 cm group (Table 3 and Fig. 2; *p* = 0.035).

**Table 3:** Expected and observed pathologic results from < 4 cm, 4–6 cm, and ≥ 6 cm adrenal masses (*p* = 0.035)

		Size			Total
		Less than 4 cm	4–6 cm	6 cm or greater	
*Pathology*					
Adrenocortical carcinoma	Count	0	0	3	3
	Expected count	1.2	1.0	0.9	3.0
Renal cell carcinoma	Count	0	0	1	1
	Expected count	0.4	0.3	0.3	1.0
Adrenal adenoma	Count	28	22	15	65
	Expected count	24.9	21.4	18.7	65.0
Metastasis	Count	0	2	2	4
	Expected count	1.5	1.3	1.2	4.0
Total	Count	28	24	21	73
	Expected count	28.0	24.0	21.0	73.0

## Discussion

Continued innovation in imaging modalities has enhanced sensitivity in identifying abnormalities of the adrenal glands. The prevalence of adrenal adenomas in the general population is estimated at 3–7%, with the overwhelming majority of those discovered incidentally demonstrating benign pathology [8]. However, because the risk of primary adrenocortical carcinoma is reported to be as high as 4.7% in patients with incidentally discovered adrenal masses, characterization and appropriate treatment remain crucial [9]. The prevalence of clinically unapparent adrenal masses at autopsy is reported at nearly 2.1%. Detection is increasing with the advent of modern imaging technology. Estimates of incidence range from 0.1% for general health ultrasound screening to 0.42% in patients evaluated for non-endocrine symptoms to 4.3% in patients previously diagnosed with cancer. This prevalence also varies with age, at < 1% for patients younger than 30 years of age and increasing to 7% in patients 70 years of age or older [10]. Overall, incidentally identified adrenal masses occur in up to 9% of CT scans, justifying the need for accurate guidelines to direct their management [11].

Advancements in imaging also allow for better characterization of adrenal masses prior to biopsy or surgical resection. Technological developments have improved the anatomic resolution, sensitivity, and specificity of cross-sectional imaging modalities, particularly MRI and contrasted CT (recommended modalities in the setting of AI), over the last 15–20 years [5]. This is fueled by the superiority of these modalities over their conventional counterparts in the accurate detection of disease. Similarly, advancements have led to an increase in data generated with more cross-sectional images per scan [12]. Improved cross-sectional imaging allows for detection of intralesional fat, water and blood enabling characterization of benign adrenal masses, such as adenomas, angiomyolipomas, cysts, granulomas and hemorrhage. Nieman et al. emphasize the importance of washout in delayed-phase CT scan due to the high prevalence of benign histology in masses with washout > 50% [13, 14]. When inconclusive, or if concerning features are noted, it may be appropriate to establish a tissue diagnosis by performing adrenal biopsy or perform definitive surgical management for potentially malignant lesions. Accurate characterization is imperative due to the potential for metastases to the adrenal gland, which may preclude surgical or radiation therapy to another primary site [11].

An additional factor contributing to increased detection is increased utilization of topographic imaging. In 2008, an estimated 60 million CT scans were performed in the United States growing at a rate of 5% per year [15]. This is astounding when compared to the 3.8% rate of increased imaging amongst Medicare patients between 1993 and 1999 and parallels the increase in technology occurring with cross-sectional imaging [16]. This widespread use of cross-sectional imaging is thought to have occurred because of ordering physicians’ attempts to address demands of both consumers and referring physicians, while providing superior patient care [15]. A significant proportion derives from a somewhat overzealous usage of cross-sectional imaging in emergency departments [17]. A 2013 study revealed that CT technology is available in 97% of emergency department (ED) visits in the United States, and is performed during 11.4% of visits, most commonly for complex abdominal pain [18]. Similarly, Bellalio et al. report an almost 60% increase in CT utilization in ED’s from 2005 to 2013 with an order for CT in 17.8% of visits. The only population that saw a decrease in utilization was children, who were not included in our study and are less likely to have adrenal incidentalomas [17]. These data are corroborated by Baloescu, who underscores that even with greater than 60 million CT scans ordered in ED’s in the United States in 2005, no decrease in morbidity and mortality was appreciated [19]. It is less likely that this increase in cross-sectional imaging is coming from primary care providers in the outpatient setting, as Weilburg et al. demonstrate the ability of a utilization management system to decrease the number of high cost imaging studies (CT, MRI, nuclear imaging, and PET) ordered in the outpatient setting from 0.43 exams per year in 2007 to 0.34 exams per year in 2013, with a cohort of around 100,000 patients [20]. Clearly, it is possible to implement systems in which the over-utilization of cross-sectional imaging is curtailed, but it remains prevalent in EDs and other clinical settings, including inpatient admissions, leading to the common discovery of AIs highlighted in our study.

With increased detection comes an increased demand for evidence-based clinical recommendations to appropriately guide health care personnel in the management of AIs. Multiple authors have demonstrated the benign nature of the majority of small adrenal masses. The AACE and AAES published guidelines a decade ago highlighting their recommendations for workup and management of such lesions. In short, they recommend clinical, biochemical, and radiologic evaluation of patients with signs of hypercortisolism, hyperaldosteronism (if hypertensive), pheochromocytoma, or malignancy. Adrenalectomy is recommended after hormonal evaluation in patients with a mass ≥ 4 cm or with malignant features on CT scan. Adrenalectomy is also recommended in smaller, less suspicious tumors in the presence of hormonal abnormalities as evidenced by aldosterone concentration/renin activity, plasma-free metanephrines and normetanephrines, and overnight 1-mg dexamethasone suppression test. Patients in whom adrenalectomy is not recommended (< 4 cm homogenous lesions with regular borders and < 10 HU on non-contrast CT) should follow surveillance guidelines, which are discussed below [5].

The 4 cm cutoff for adrenalectomy is well-established and perhaps too-readily accepted by those outlining guidelines for the management of AIs. Mantero et al. reported that 4 cm had a greater sensitivity (93%) for detection of ACC when compared to 5 cm (81%) and 6 cm (74%), respectively. However, their 2000 study was limited to an Italian population that included only 1004 patients and the issue of size’s ability to predict benign or malignant pathology has been revisited little in the past two decades [21]. Sturgeon et al. evaluated 457 patients with ACC and 47 patients with adrenal adenomas, establishing the sensitivity of tumor size in predicting malignancy to be 96% for tumors ≥ 4 cm, 90% for tumors ≥ 6 cm, and 77% for tumors ≥ 8 cm. Based on their data, at a threshold of ≥ 4 cm, the likelihood of malignancy doubles (to 10%) [22]. Cutoffs ranging from 4 to 6 cm have been proposed for surgical excisional of incidentally-discovered adrenal masses. A recent analysis of 2219 patients by Kahramangil et al. shows ACC rates of 0.1%, 2.4%, and 19.5% in patients with masses < 4 cm, 4–6 cm, and > 6 cm, respectively, with an optimal cutoff of 4.6 cm. In addition, analysis by Hounsfield (HU) density on non-contrast CT showed ACC risk of 0%, 0.5%, and 6.3% for masses of < 10, 10–20, and > 20 HU, respectively. In addition, male sex and > 0.6 cm/year growth rate were independent predictors of ACC [7]. Similarly, Birsen et al. [23] describe development of a scoring system to determine the probability of an adrenal mass representing ACC based on size and HU. Of their 157 patients, seven without hormonal secretion had ACC on final pathology; of these, only a single ACC measured < 6 cm [23]. Due to increasing risk of malignancy with those > 4 cm, authors readily accept 4 cm as the cutoff. However, our data suggests that this cutoff may be overly-cautious and subjects patients to unnecessary morbidity that accompanies adrenalectomy.

Along with a conservative approach to management comes a need to clarify ongoing surveillance imaging and laboratory evaluation intervals. This is due to a 17%, 29%, 47% risk of AIs converting to functional status and a 6%, 14%, 29% risk of increasing in size at 1, 2, and 5 years, respectively [5]. Current guidelines vary, with most suggesting follow-up guided by clinical judgment and the presumed cause of the mass based on initial workup. National Institute of Health (NIH) data suggests that for masses that are benign-appearing (< 10 HU; washout > 50%), small (< 3 cm), and completely non-functioning, imaging and biochemical reevaluation at 1–2 years (or more) is appropriate, with subsequent follow-up only if the clinical picture changes; the risk of malignancy or subsequent hyperfunction is almost nonexistent though reported conversion to ACC exists in recent literature [24]. For indeterminate lesions, repeat evaluation for growth after 3–12 months is appropriate. Subsequent testing should occur earlier for lesions with increasing size, and later for those with no change [13]. The AAES extends the criteria for “small” AIs to include those < 4 cm and they better outline surveillance recommendations to include radiographic reevaluation at 3 to 6 months, then annually for 1–2 years. Similarly, they suggest hormonal evaluation annually for up to 5 years [5]. Repeat screening for hyperaldosteronism is not perceived to be beneficial, but most authors recommend screening for catecholamine and cortisol excess for at least four years due to the similar appearance of pheochromocytomas to lipid-poor adenomas on CT scan. This is one instance in which clinical judgment can be used to guide surveillance as most pheochromocytomas grow over time suggesting that further imaging may not be indicated in the setting of a stable mass [13].

The above algorithms include 1–3 radiological assessments in the first two years with reconsideration of surgical excision with 0.5–1 cm of growth during follow up. Corwin et al. [25] assessed 131 adrenal masses, 26 of which were found to be malignant and 121 adenoma. Of these, all malignant nodules increased in size during follow-up, with a mean growth of 5.8 cm/year. A growth rate of 3 mm/year distinguished adenomas from malignant nodules with sensitivity and specificity of 100% [25]. Repeating the same imaging modality (generally CT scan) is encouraged to assess for changes in the mass [6]. The American College of Radiology better defines imaging protocols, including measurement of density and contrast washout, as well as the importance of clinical correlation when determining surveillance regimens for AIs < 4 cm. Specifically, in 2–4 cm AIs, an adrenal CT protocol should be used at diagnosis to better characterize the lesion, while lesions < 2 cm may have an adrenal CT protocol at one year to document stability. Chomsky-Higgins et al. performed a cost-effectiveness analysis of surveillance of adrenal masses 1–4 cm with an initial normal workup including non-contrasted CT showing a mass < 10 HU, no suspicious features, and negative hormonal evaluation. They determined it is most effective to perform a single follow up at one year with non-contrasted CT and biochemical evaluation [26]. It may be reasonable to consider excision in those adrenal masses with indeterminate radiologic features that grow at least 0.8 cm during 3–12 month follow-up [14]. Post-excision surveillance for AIs > 4 cm remains poorly defined and is based on the histological diagnosis at the time of extirpation [27]. Although recommendations for radiologic follow-up of nonfunctional adrenal masses vary based on the organization, it is important to consider that frequent adrenal imaging is associated with additional cost, anxiety, and exposure to radiation, which may theoretically induce cancer at an estimated rate similar to the chance of developing adrenal malignancy [14].

This study provides insight into adrenal masses, particularly ACC, in a rural state with the highest rate of smoking and the second highest rate of ACC in the country. In fact, West Virginia has been used as a model for the establishment of smoking as a risk factor for ACC, making it an appropriate site for evaluation of the workup, diagnosis, treatment, and pathologic outcomes of adrenal masses, even in the setting of lower overall surgical case numbers [28]. Limitations of this study include its retrospective nature, which has the potential to introduce biases in data collection and analysis. Although it is a multi-institutional study, both institutions are within the same geographic region with a notably homogenous population, limiting its applicability to wider groups. Also, the lack of surveillance data within the population limits our ability to detect recurrence amongst participants and growth of non-operative AIs. Similarly, surgical patients are the only subjects included, limiting the ability to assess cancer risk in a more specific population of patients on active surveillance who undergo resection following AI growth. However, the above study design achieves the goal of assessing pathological outcomes in patients undergoing adrenalectomy for incidentally detected adrenal masses. Addition of patients to our study data from institutions with more diverse populations in terms of race and age as well as surveillance data provide future directions for this research.

## Conclusions

Regarding surgical excision of an AI, the decision to operate is difficult in a patient with no known cancer history, a negative hormonal evaluation, and inconclusive radiographic characteristics. The findings of this study suggest that a 6 cm threshold for surgical excision may be considered to avoid removal of benign lesions while maintaining an acceptable sensitivity for adrenocortical carcinoma. Significant overlap exists between guidelines for surveillance of adrenal masses. Further investigation is warranted to establish evidence-based recommendations which more thoroughly outline surveillance protocols of AIs.

## Data Availability

The datasets used and/or analyzed during the current study are available from the corresponding author on reasonable request, our database is not currently de-identified preventing its provision with the accompanying manuscript.

## Abbreviations

AI: Adrenal incidentaloma
ACC: Adrenocortical carcinoma
AACE: American Association of Clinical Endocrinologists
AAES: American Association of Endocrine Surgeons
WVU: West Virginia University
CAMC: Charleston Area Medical Center
